# From Functional Food to Therapeutic Prospect: Mechanistic Study of Gypenoside XVII in HeLa Cells

**DOI:** 10.3390/molecules31020214

**Published:** 2026-01-08

**Authors:** Sayed Sajid Hussain, Muhammad Maisam, Shoaib Younas, Feng Wang, Weijie Li

**Affiliations:** 1 School of Food Science and Biological Engineering, Hefei University of Technology, Hefei 230009, China; 2 School of Health Science and Engineering, University of Shanghai for Science and Technology, Shanghai 200093, China

**Keywords:** gypenoside XVII, HeLa, cell cycle analysis, Hoechst 33342, cell apoptosis, Western blotting

## Abstract

Cervical cancer remains a prominent cause of cancer-related mortality among women worldwide because of chronic infection with high-risk human papillomavirus (HPV) and disparate access to prevention and treatment. The current research evaluates the anticancer activity of Gypenoside XVII, a bioactive saponin of *Gynostemma pentaphyllum*, in HeLa cells as a model of cervical cancer. MTT, Annexin V-PI, and Hoechst 33342 assays showed dose-dependent growth inhibition with typical apoptotic morphology. Flow cytometry revealed G_0_/G_1_ cell-cycle arrest, while pathway interrogation revealed participation of mitochondrial and death-receptor cascades, in agreement with caspase-9 and caspase-8 activation, respectively. Collectively, these findings position Gypenoside XVII as a natural-product bioactive with potential both as an anticancer lead and as a functional-food ingredient, deserving of further preclinical development.

## 1. Introduction

Cancer has been one of the biggest challenges in the world of healthcare; it accounts for more deaths than stroke and any type of cardiovascular disease. The World Health Organization (https://www.who.int/cancer/prevention/en/, accessed on 12 August 2025) indicated that it is responsible for the largest number of deaths in the world. There has been an estimate that because of the aging population and demographics, cancer cases will increase across the world, and in 2050, there will be a projected 35 million cases of cancer reported in the less developed countries of the world. According to the Global Cancer Report 2022, there are about 20 million cases and 9.7 million deaths caused by cancer [1]. The high number of deaths in Asia and Africa is attributed to the fact that the cancer case-fatality ratios are high, owing to the distribution of cancer types and the delay in diagnosing cancer cases. Although Europe represents only 9.6% of the global population, it accounts for 22.4% of new cancer cases and 20.4% of cancer-related deaths, highlighting a significant regional disease burden influenced by cancer prevalence and healthcare constraints [2]. When considering female cancers, cervical cancer is a major public health problem and accounts for a substantial proportion of cancer deaths in females [3]. In 2023, there were 13,960 newly reported cases and 4310 deaths due to cervical cancer. Moreover, estimates suggest that by 2026, there will be 528,000 newly reported cases of cervical carcinoma, of which about 85% will be from developing nations [4]. Even with advances in prevention, screening, management, and resistance, the lack of accessibility to healthcare is hampering efficient management of cervical carcinoma, thus prompting a quest for alternative and complementary therapeutic strategies.

Varying treatment modalities have been utilized for cancer treatment; yet, research for more effective, low-cost, and non-toxic therapeutic agents continues to gain emphasis. In this respect, medicinal plants have been gaining considerable attention because of their rich bioactive constituents and established medicinal applications. *Gynostemma pentaphyllum* (Thunb.) Makino, which has been referred to in Chinese literature by the name “Jiao Gu Lan,” is an evergreen scaling plant of the *Gynostemma* species (family *Cucurbitaceae*), which grows in substantial portions of Northeastern to Southeastern Asia [5,6,7,8]. Apart from its medically significant properties, *G. pentaphyllum* has been employed in China for years as a functional food ingredient in the form of tea drinks, beverages, noodles, and dietary supplements. Domestically, it grows in portions of mainland China situated south of the Yangtze River, along with the Qinling Mountains [9,10]. It has been employed in folk medicine for treating a range of conditions like hematuria, pharyngitis, edema, inflammation, and trauma [11].

Globally, *G. pentaphyllum* has attracted intense attention and has been sold in different forms like powders, teas, tablets, capsules, and oral liquids in the USA, China, and various Asian and European nations [12,13,14]. Indeed, various studies have revealed its vast potential in treating different diseases like cancer, bacterial infections, aging, chronic fatigue syndrome, ulcers, hypolipidemia, and immunoregulators [15,16,17,18]. In the same vein, different phytochemical studies have also isolated numerous bioactive compounds like saponins [19], carotenoids [20], flavonoids [9], chlorophylls [21], lignins [22], and polysaccharides [23]. Among them, saponins, also known as “gynosaponins” or “gypenosides”, are specifically noteworthy, with more than 280 different molecules discovered thus far. Gypenosides are specifically recognized to be the major bioactive fractions of *G. pentaphyllum* responsible for different biologically vast activities exerted by this plant species [24]. Although anticancer activities of different gypenosides have already been studied using various cancer cell lines such as Eca-109 (esophageal cancer), SW620 (colon cancer), SAS (oral cancer), and cervical epidermoid carcinoma cells [25,26,27], the antitumor activities and mechanisms of action of different individual gypenosides are still unclear. Additionally, little research has been conducted on them regarding their anticancer properties, and specifically, little has been done on the anticancer properties of Gypenoside XVII [28,29].

In the current study, we investigated the in vitro anticancer effects of Gypenoside XVII on human cervical carcinoma HeLa cells regarding its influence on cell proliferation, cell-cycle progression, and apoptosis-related signaling pathways. This study also explored mechanistic insights into both intrinsic and extrinsic apoptotic pathways, thereby providing initial evidence that Gypenoside XVII might be considered a biologically active natural compound for further preclinical investigation.

## 2. Results

### 2.1. Inhibition Effect of Gypenoside XVII on HeLa Cell Proliferation

**Figure 1:**
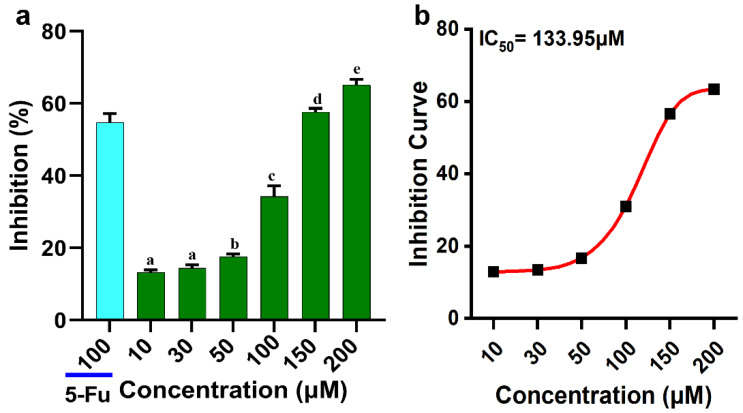
HeLa cell growth is inhibited by 5-FU and Gypenoside XVII. (**a**) The dose–response inhibition curve of Gypenoside XVII on HeLa cells, featuring the IC_50_ value, indicates the concentration at which Gypenoside XVII attains 50% inhibition. (**b**) The rate of inhibition, with a concentration of 100 µM 5-FU serving as a positive internal control. All treated cells underwent incubation for 24 h. Different letters (a–e) above the bars show statistical differences among groups at the *p* < 0.05 level and were determined using one-way ANOVA followed by post hoc analysis.

The inhibitory effect of Gypenoside XVII on the proliferation of HeLa cells was determined by MTT assay. As shown in Figure 1a, there was an inhibition effect on cell viability in response to an increase in the concentration of Gypenoside XVII on HeLa cells for 24 h. This indicated there is a dose-dependent cytotoxic effect on the proliferation of HeLa cells.

The half-maximal inhibitory concentration (IC_50_), which is described as the concentration needed to inhibit 50% of cell growth, was calculated using curve fitting and found to be 133.39 µM Figure 1b. The inhibition rate of 55.24% for 5-fluorouracil (5-FU), a chemotherapeutic agent, was also measured under the same experimental conditions that were employed as a positive control.

### 2.2. Effect of Gypenoside XVII on Cell Cycle

**Figure 2:**
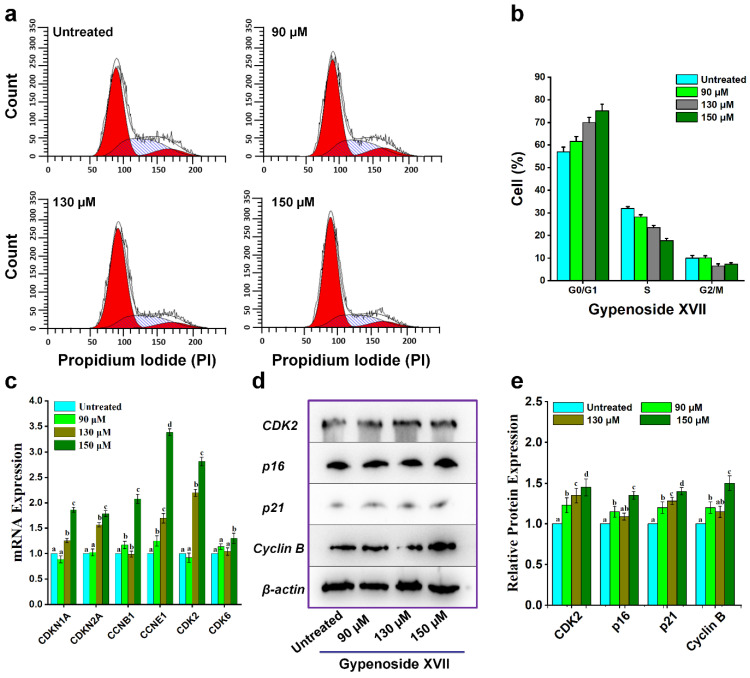
Cell cycle distribution was examined using flow cytometry. (**a**) Distribution of HeLa cells among the G_0_/G_1_, S, and G2/M phases following treatment with different doses of Gypenoside XVII. (**b**) Percentage of cells in each phase of the cell cycle across various treatment conditions. One-way ANOVA was utilized for statistical analysis. (**c**–**e**) Exhibit HeLa cells treated with and without Gypenoside XVII were subjected to RT-PCR and Western blotting to assess the expression levels of cell cycle-related genes and proteins. (**c**) RT-PCR data indicating the expression levels of cell cycle-associated genes in HeLa cells subjected to treatment with and without Gypenoside XVII. Western blot analysis (**d**,**e**) demonstrating the impact of Gypenoside XVII on cell cycle-associated proteins in HeLa cells. Different letters (a–d) above the bars show statistical differences among groups at the *p* < 0.05 level and were determined using one-way ANOVA followed by post hoc analysis.

Analysis of Gypenoside XVII-induced influence on the distribution of HeLa cells at various phases of the cell cycle was performed using flow cytometry Figure 2a. There was a significant increase in the number of cells in the G_0_/G_1_ phase following treatment with Gypenoside XVII for 24 h, concomitant with a reduced number of cells in phases S and G_2_/M compared with the control group. There was a definite concentration-dependent arrest of cells in the G_0_/G_1_ phase after treatment with increasing concentrations of Gypenoside XVII. This was evident as the relative value of the G_0_/G_1_ phase increased from 57.70% in the control to 61.59%, 70.01%, and 75.12% after treatment with concentrations of 90 µM, 130 µM, and 150 µM, respectively. At the same time, the value of the S and G_2_/M phases decreased. The distribution of cells at various phases of the cell cycle is presented in Figure 2b.

In order to further shed light on the molecular basis behind G_0_/G_1_ arrest, the expression levels of G_0_/G_1_ cell cycle-related genes and proteins were further analyzed by RT-qPCR and Western blot analysis (Figure 2c–e) and uncropped images are available in Supporting Information (Figure S1a–e). Gypenoside XVII induced G_0_/G_1_ cell cycle regulating gene expression in a concentration-dependent manner, including *CCNB1*, *CCNE1*, *CDK2*, and *CDK6* (showing a statistically significant increase at higher concentrations), as well as *CDKN2A* and *CDKN1A* at the mRNA expression level. Corresponding alterations in protein expression were also observed, further supporting the transcriptional findings.

Collectively, these findings showed that Gypenoside XVII inhibits cell cycle progression and arrests HeLa cells in the G_0_/G_1_ phase by modulating important regulators of cell cycle progression.

### 2.3. Effect of Gypenoside XVII on HeLa Cell Apoptosis

Based on our previous investigation, which revealed that extracts (Stevenleaf) of *G. pentaphyllum* could induce apoptosis in human hepatoma cells HepG2 [30], we evaluated the ability of Gypenoside XVII from *G. pentaphyllum* to trigger apoptotic cell death in cervical carcinoma HeLa cells. For this purpose, HeLa cells were exposed to gradually increasing doses of Gypenoside XVII (0, 90, 130, and 150 µM) for 24 h, and the cells were then stained with Annexin V-FITC/PI and analyzed.

**Figure 3:**
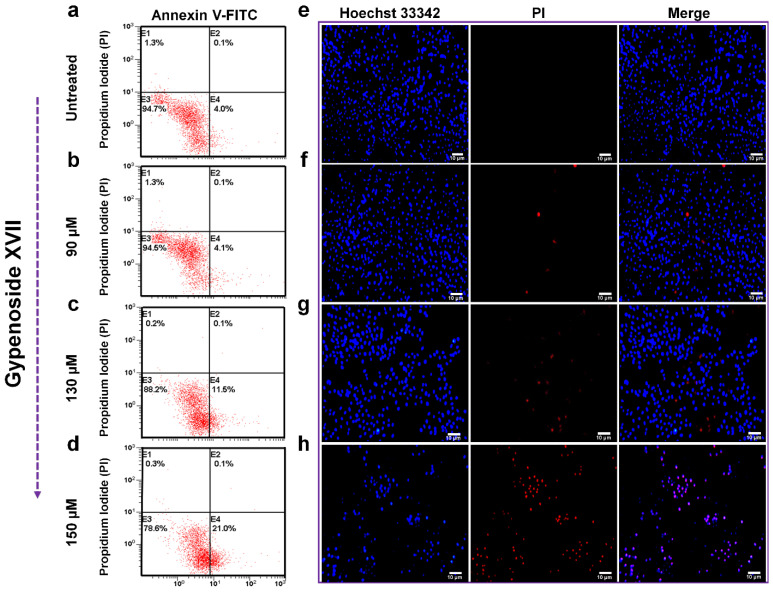
Annexin V-PI labeling followed by flow cytometry (FCM) analysis to assess apoptosis, and Hoechst 33342 staining to detect apoptotic cell morphology, were used to assess apoptosis in HeLa cells treated with Gypenoside XVII for 24 h. Results are shown for the subsequent treatment groups: (**a**) Untreated; (**b**) 90 µM; (**c**) 130 µM; (**d**) 150 µM for Annexin V-PI labeling, and (**e**) Untreated; (**f**) 90 µM; (**g**) 130 µM; (**h**) 150 µM. Scale bar: 10 µM for Hoechst 33342 staining.

As shown in Figure 3a–d, there was a concentration-dependent increase in apoptotic cell numbers in Gypenoside XVII-treated cells compared to untreated controls. The percentage of cells was significantly increased in the early apoptotic stage (Annexin V+ and PI negative).

Apoptotic induction was further validated by Hoechst 33342/PI staining for nuclear morphology (Figure 3e–h). Although there were no apparent intensity changes in Hoechst staining, subjective analysis revealed occasional chromatin condensations in Gypenoside XVII-treated cells. There were also dose-dependent increases in the red fluorescent signals of PI, which indicate cells undergoing late apoptosis or secondary necrosis [31,32,33].

These findings, together, indicate that apoptosis mediated by Gypenoside XVII occurs in HeLa cells in a dose-dependent manner. It is important to note that the quantitative analysis of apoptosis was mainly performed using Annexin V-FITC/PI flow cytometry, and the results were supported qualitatively by Hoechst/PI staining.

### 2.4. Effect of Gypenoside XVII on Mitochondrial Pathway

**Figure 4:**
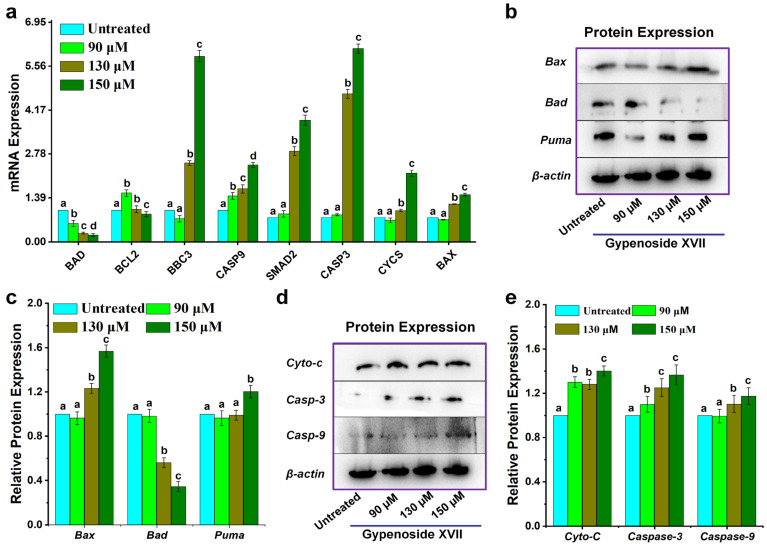
The expression levels of genes and proteins linked to the mitochondrial pathway were examined in HeLa cells treated with Gypenoside XVII at concentrations of 90 µM, 130 µM, 150 µM, and 0 µM (untreated). (**a**) Expression levels of mRNA for genes associated with the mitochondrial pathway following 24 h of Gypenoside XVII administration. (**b**–**e**) Protein expression levels of mitochondrial pathway-associated proteins in HeLa cells treated with Gypenoside XVII. Different letters (a–d) above the bars show statistical differences among groups at the *p* < 0.05 level and were determined using one-way ANOVA followed by post hoc analysis.

To investigate whether Gypenoside XVII activates mitochondrial-mediated apoptosis, the expression of key genes and proteins associated with the intrinsic apoptotic pathway was examined in HeLa cells. Following 24 h treatment with Gypenoside XVII, RT-qPCR analysis revealed a significant, concentration-dependent upregulation of *CASP3*, *CASP9*, *CYCS*, *BAX*, *BBC3*, and *SMAD2* mRNA levels compared with untreated controls (Figure 4a). In contrast, the expression of the anti-apoptotic genes *BAD* and *BCL2* was markedly reduced.

These transcriptional changes were further supported by Western blot analysis, which demonstrated corresponding alterations in protein expression levels of mitochondrial pathway-associated markers following Gypenoside XVII treatment (Figure 4b–e), and uncropped images are available in Supporting Information (Figure S2a–f). The consistency between mRNA and protein expression profiles suggests that Gypenoside XVII modulates key components of the mitochondrial apoptotic pathway in HeLa cells.

### 2.5. Effect of Gypenoside XVII on Death Receptor Pathway

**Figure 5:**
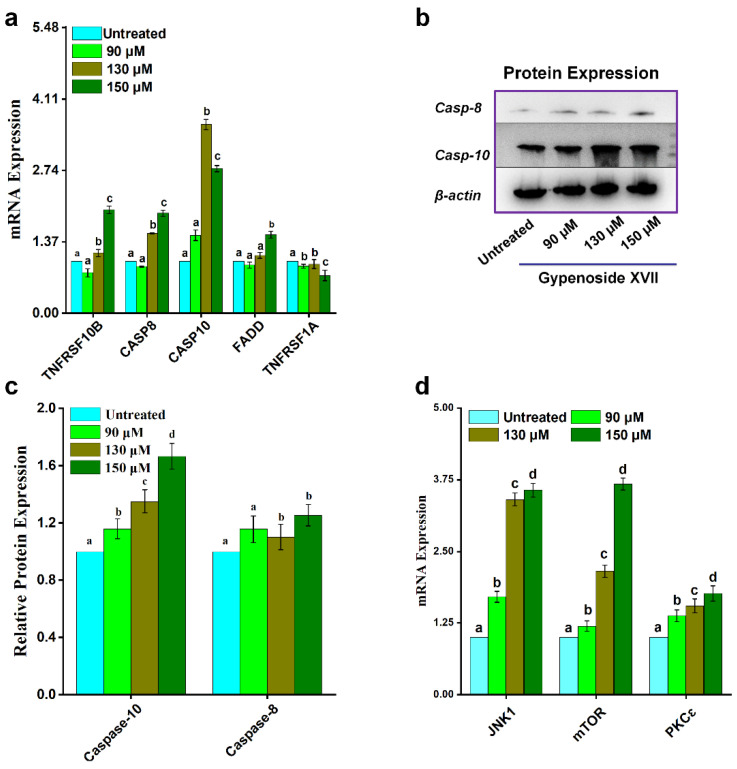
Gypenoside XVII’s effects on HeLa cells include changes in the death receptor pathway and other genes linked to apoptosis. (**a**) mRNA expression levels of genes associated with the death receptor pathway. (**b**) Protein expression levels of the identical pathway. (**c**) Gene expression levels of additional apoptosis-related pathways in HeLa cells subjected to varying doses of Gypenoside XVII. (**d**) mRNA expression levels of genes associated with other apoptotic related pathway. Different letters (a–d) above the bars show statistical differences among groups at the *p* < 0.05 level and were determined using one-way ANOVA followed by post hoc analysis.

To assess the involvement of the extrinsic apoptotic pathway, the expression of key death receptor-associated genes and proteins was analyzed in HeLa cells following 24 h treatment with Gypenoside XVII. RT-qPCR results demonstrated a significant upregulation of *FADD*, *CASP8*, *CASP10*, and *TNFRSF10B* mRNA levels compared with untreated controls, whereas *TNFRSF1A* expression was markedly reduced (Figure 5a).

Consistent with these transcriptional changes, Western blot analysis confirmed corresponding alterations in protein expression levels of extrinsic pathway-related markers after Gypenoside XVII treatment (Figure 5b,c), (Figure S3a,b). These findings indicate that Gypenoside XVII modulates key components of the death receptor-mediated apoptotic pathway in HeLa cells.

### 2.6. Effect of Gypenoside XVII on Other Apoptosis-Related Genes

In addition to its effects on the intrinsic and extrinsic apoptotic pathways, the influence of Gypenoside XVII on other apoptosis-related genes was examined at the transcriptional level. RT-qPCR analysis showed that treatment of HeLa cells with Gypenoside XVII resulted in a significant upregulation of *JNK1*, *mTOR*, and *PKCɛ mRNA* expression compared with untreated controls (Figure 5d).

These findings suggest that Gypenoside XVII modulates the expression of additional signaling molecules associated with apoptosis-related regulatory pathways in HeLa cells.

## 3. Discussion

Cancer is still a significant global health issue that demands a ceaseless effort towards the development of anticancer drugs that are more selective and less toxic. Natural products have long been a valuable source of bioactive compounds [1,34]. There is growing interest in plant-derived saponins due to their wide range of pharmacological activities. Based on these considerations, Gypenoside XVII, a saponin isolated from *Gynostemma pentaphyllum,* was studied for antiproliferative and pro-apoptotic activity on human cervical cancer HeLa cells. Although cytotoxicity to normal cervical cells was not evaluated in this study, the dose-dependent decrease in viability observed across the MTT assay would suggest that Gypenoside XVI acts in a non-indiscriminate manner, and its selectivity in normal epithelial models deserves further pursuit.

Dysregulation of the cell cycle has been widely implicated in cancer progression, and cell cycle regulation represents an attractive therapeutic approach. Flow cytometric analysis confirmed that Gypenoside XVII treatment significantly increased the number of HeLa cells in the G_0_/G_1_ phase, suggesting arrest at this critical checkpoint. The G_1_ phase of the cell cycle is regulated by Cyclin–Cyclin-Dependent Kinase (CDK) complexes, such as Cyclin D/CDK4/6 and Cyclin E/CDK2, which control DNA synthesis and mitotic events [35]. The action of CDK inhibitors, such as p21 and p16, represented a critical step in regulating these complexes and inducing G_0_/G_1_ arrest. Previous studies have confirmed that p21 binds to Cyclin/CDK complexes to inhibit CDK activity, suppress cell cycle progression, and facilitate some degree of tumor inhibition [36]. Similarly, p16 suppressed the activity of CDK4/CDK6, prevented the phosphorylation of the Retinoblastoma Protein, and arrested the G_1_ phase of the cell cycle [37,38,39]. Notably, an increase in the expression of p16, p21, and Cyclin E in Gypenoside XVII-treated cells suggests that the suppression of HeLa cell proliferation by Gypenoside XVII is mediated by G_0_/G_1_ regulatory molecules. Apart from the inhibition of the cell cycle, the induction of apoptosis is another important mechanism of the antitumor effects of various chemotherapeutic drugs. Flow cytometric analysis using Annexin V-FITC/PI staining demonstrated that Gypenoside XVII significantly increased the proportion of apoptotic cells, particularly in the early apoptotic phase. Notably, after 24 h of treatment, the percentage of early apoptotic cells increased from 4.1% in untreated controls to 21.0% in treated cells, indicating effective initiation of programmed cell death. Indeed, the relatively high IC_50_ value obtained in this study is consistent with the exploratory evaluation of the derivative of a functional food saponin, and not with an optimized chemotherapeutic agent, and reflects the preliminary, mechanistic nature of the present investigation.

To better understand the involvement of apoptosis mechanisms, the extrinsic and intrinsic pathways were analyzed. The extrinsic apoptosis pathway was initiated by the increased expression of *FADD*, *Caspase-8*, *Caspase-10*, and *DR5*, accompanied by the downregulation of *TNF-R1*. Death receptor signaling begins when ligands bind to members of the TNF receptor superfamily, resulting in the recruitment of adapter molecules like *FADD* and the assembly of the Death-Inducing Signaling Complex (DISC), ultimately leading to caspase activation and apoptosis [40,41]. The observed changes in transcriptional and protein expression levels suggested that Gypenoside XVII modulated key components of this pathway in HeLa cells.

The intrinsic (mitochondrial) pathway of apoptosis is mainly governed by the BCL-2 family of proteins, which encompasses both pro-apoptotic and anti-apoptotic proteins [42,43]. Among these, the BH3-only molecule Puma plays a crucial role in initiating outer mitochondrial membrane permeabilization following Bax activation [43]. Consistent with these notions, our data demonstrated upregulation of Bax and Puma, downregulation of BCL-2 expression, and elevated levels of cytochrome c, Caspase-9, and Caspase-3 as a consequence of Gypenoside XVII treatment. These expressions manifested a sequential activation of the intrinsic pathway of apoptosis.

While induction of apoptosis and cell cycle arrest are common outcomes of in vitro anticancer studies, the present work was intended as a preliminary mechanistic study rather than a claim of immediate translational relevance. To our knowledge, this study represents the first systematic assessment of the anticancer properties of Gypenoside XVII in human cervical cancer cells, revealing its coordinated modulation of mitochondrial (intrinsic) and death receptor-mediated (extrinsic) apoptotic pathways as well as G_0_/G_1_ cell cycle arrest. Instead of reflecting the activation of a single generalized pathway, the molecular changes observed suggested a multifaceted cellular response to Gypenoside XVII.

**Figure 6:**
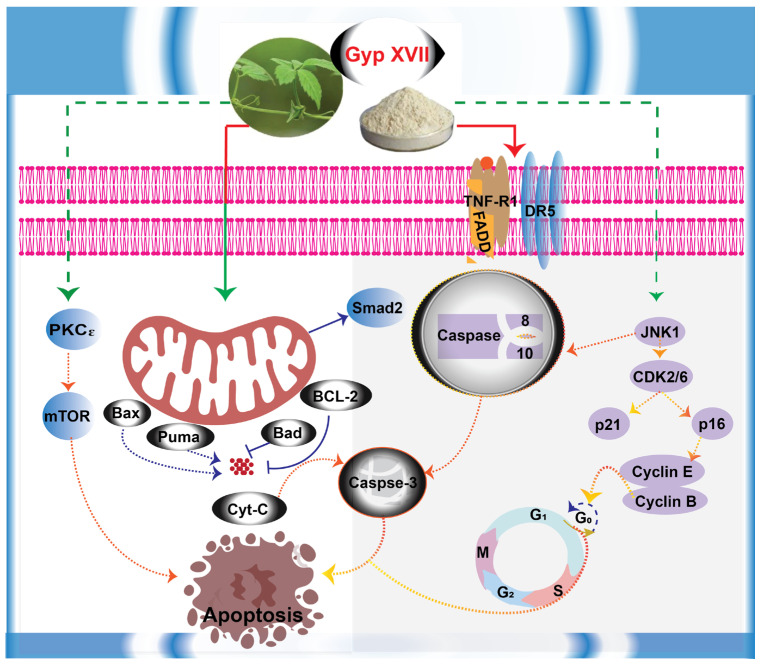
Proposed molecular mechanism of Gypenoside XVII treatment in HeLa cells.

Overall, the current study exhibited that Gypenoside XVII possessed antiproliferative activities in HeLa cells through G_0_/G_1_ phase cell cycle arrest and apoptosis triggered by the regulation of both intrinsic and extrinsic apoptosis pathways. Although these pathways are well-known regulators of apoptosis triggered by antitumor compounds in vitro studies, the current study was able to provide a mechanism behind the biological activities of Gypenoside XVII in cervical cancer cells. A graphical summary of the proposed mechanism of Gypenoside XVII in acting as an antitumor compound through a combination of G_0_/G_1_ phase cell cycle arrest and apoptosis is shown in Figure 6.

## 4. Experimental Method

### 4.1. Chemicals and Reagents

Gypenoside XVII was obtained from Chengdu Must Bio-Technology Co., Ltd. (Chengdu, China) PR China (molecular weight: 947.15 g/mol; purity: 99.6%). All chemical reagents, including ribonuclease-A, Tris HCl, potassium phosphate, and propidium iodide (PI), were sourced from Shanghai Sinopharm Chemical Reagent Co., Ltd. (Shanghai, China). Penicillin–streptomycin, glutamine, fetal bovine serum (FBS), Dulbecco’s Modified Eagle’s Medium (DMEM), and trypsin-EDTA were purchased from Invitrogen (Carlsbad, CA, USA). Specific inhibitors of caspase-3 and caspase-8 were acquired from MedChemExpress (Monmouth Junction, NJ, USA).

### 4.2. Cell Preparation

A human cervical cancer cell line called HeLa cells was purchased from Shanghai Wei Atlas Biological Technology Co., Ltd. (Shanghai, China). All experiments were carried out under observation. The DMEM media supplemented with 6 mM glutamine, 100 U/mL penicillin, 100 µg/mL streptomycin, and 10% FBS was used to culture the cells. To promote the best possible adhesion to the plates, the cells were incubated for 24 h at 37 °C in an atmosphere with 5% CO_2_ and 95% air.

### 4.3. MTT Assay

After HeLa cells were seeded and incubated for 12 h, 100 µL of the cell suspension (5 × 10^4^ cells/well) was added to each well of a 96-well plate. 50 µM 5-fluorouracil (5-FU) served as a positive control before gypenoside XVII was given at various dosages (0 µM, 10 µM, 30 µM, 50 µM, 100 µM, 150 µM, and 200 µM). For a whole day, the plates were incubated. Following the addition of 20 µL of 5 mg/mL MTT reagent to each well, the plates were incubated for an additional 4 h. The generated formazan crystals were then dissolved by using 150 µL of DMSO. At 570 nm, the absorbance was measured with the help of a microplate reader [30].

### 4.4. Cell Cycle Analysis

Following that, six-well plates were seeded with 3 × 10^5^ HeLa cells. After 12 h, several quantities of Gypenoside XVII were added: 0 µM, 70 µM, 90 µM, 110 µM, 130 µM, and 150 µM. Trypsinization was used to harvest the treated and normal cells following a 24-h incubation period at 37 °C in a CO_2_ incubator. After being rinsed with 1× phosphate-buffered saline (PBS), the cells were gradually fixed by adding 70% ethanol and kept at 4 °C for the entire night. After that, the cells were reconstituted in PI solution and allowed to sit in the dark for thirty minutes. In the end, the samples were examined using flow cytometry (BD Accuri C6, San Jose, CA, USA) with a 488 nm excitation wavelength [44].

### 4.5. Cell Apoptosis

Cell apoptosis was assessed by Annexin V-FITC double staining (Beyotime, Shanghai, China) in agreement with the manufacturer’s instructions. Following the experiment, treated cells at a concentration of 1 × 10^6^ cells/mL were resuspended in 1× Annexin binding solution. After treating the cells with 5 µL of Annexin V-FITC and 10 µL of PI, they were incubated at 2–8 °C for 15 and 5 min, respectively. Flow cytometry (FCM) was then used to identify apoptotic cells [45].

### 4.6. Measurement of Apoptotic Morphology

We used Hoechst 33342-PI staining to analyze the apoptotic morphology of HeLa cells. Gypenoside XVII was administered to HeLa cells (1 × 10^6^ cells/mL) at concentrations of 0 µM, 90 µM, 130 µM, and 150 µM. The cells were incubated for 24 h, then washed with 1× PBS and stained with Hoechst 33342 and 5 µL of PI staining solution. Before analysis, the samples were washed twice with 1× PBS after being incubated for 20 min. Finally, laser-confocal microscopy (Carl-Zeiss Microscopy GmbH, Jena, Germany) was used to detect red and blue fluorescence.

### 4.7. RT-qPCR Analysis

As highlighted in our previous studies [46], HeLa cells were subjected to treatment with different concentrations of Gypenoside XVII (0 µM, 90 µM, 130 µM, and 150 µM) to measure the expression of genes associated with the cell cycle and apoptosis. Whole RNA was extracted with RNAiso Plus reagent (TaKaRa, Dalian Company, Ltd., Dalian, China) in accordance with the manufacturer’s guidelines. The RNA was further processed to eliminate DNA and reverse-transcribed into cDNA utilizing the Takara Reverse kit. Quantitative PCR (qPCR) was conducted utilizing the LightCycler 96 System (Roche, Basel, Switzerland), with β-actin as the internal control. The nucleotide sequences employed in this study are specified in Table 1.

### 4.8. Western Blotting Analysis

HeLa cells were cultivated for 24 h after being seeded at a density of 1 × 10^6^ cells per well. For a full day, cells were exposed to several doses of Gypenoside XVII (0 µM, 90 µM, 130 µM, and 150 µM). Following the manufacturer’s instructions, proteins were isolated using a protein extraction kit. The samples were mixed with 2× loading buffer after protein measurement, and they were then heated for 10 min. Using 10% SDS-PAGE electrophoresis (Rebio, Shanghai, China), the target proteins were separated and then moved to a polyvinylidene difluoride (PVDF) membrane (Millipore, Darmstadt, Germany). Using primary antibodies (Cell Signaling Technology, Danvers, MA, USA), the membrane was treated with 5% skim milk for two hours at room temperature and then overnight at 4 °C. Secondary antibodies were subsequently given. The internal reference was β-actin. ECL luminescence was used to identify protein bands, and grayscale imaging was then used for analysis (Transgen, Beijing, China) [29].

### 4.9. Statistical Analysis

SPSS 22.0 software (SPSS, Inc., Chicago, IL, USA) was used for data analysis. One-way analysis of variance (ANOVA) was employed to identify differences between Gypenoside XVII treatment groups, with statistical significance set at *p* < 0.05.

## 5. Conclusions

This research provided insights into the in vitro anticancer effects of Gypenoside XVII, a bioactive saponin extracted from *Gynostemma pentaphyllum*, on HeLa cervical carcinoma cells. Gypenoside XVII inhibited cell proliferation, induced G0/G1 cell cycle arrest, and triggered apoptotic cell death through a comprehensive regulation of both intrinsic and extrinsic apoptotic pathways. Based on our findings, this research made a valid contribution to understanding the anticancer effects of Gypenoside XVII and shed light on its relevance as a bioactive saponin beyond its traditional application as a functional food ingredient. Even though our research is a purely in vitro experiment with limited applicability and relevance to practical anticancer therapy, our findings suggest that Gypenoside XVII could be investigated as a lead bioactive compound for comprehensive anticancer treatment in various in vivo models. In general, further research is required to clearly delineate the pathway hierarchy and target specificity, including the regulation of the BCL-2 family proteins, caspase activation cascades, and death receptor signaling, and to explore combination strategies and formulation approaches that may enhance bioavailability and therapeutic efficacy. These collective efforts will help define both the therapeutic possibilities and limitations of Gypenoside XVII and further its evaluation beyond mechanistic proof-of-concept in cervical cancer research.

## Figures and Tables

**Table 1:** Primers for RT-PCR.

Genes	Primers	Sequences	Genes	Primers	Sequences
*Caspase-3*	Forward	TGGACTGTGGCATTGAGAA	*FADD*	Forward	GGGAAGAAGACCTGTGTGCA
	Reverse	CAGGTGCTGTGGAGTATGCA		Reverse	ATTCTCAGTGACTCCCGCAC
*CDKN1A*	Forward	GCGGAACAAGGAGTCAGACA	*CCNE1*	Forward	GGATTATTGCACCATCCAGAGGCT
	Reverse	GAACCAGGACACATGGGGAG		Reverse	CTTGTGTCGCCATATACCGGTCAA
*CDKN2A*	Forward	CTTCCTGGACACGCTGGT	*CCNB1*	Forward	CTGCTGGGTGTAGGTCCTTG
	Reverse	ATCTATGCGGGCATGGTTACT		Reverse	TGCCATGTTGATCTTCGCCT
*CYCS*	Forward	AGGAGGTGGAGGCAAAGGTA	*PKC* *ɛ*	Forward	TGCCCCACAAGTTCGGTATC
	Reverse	ATATTTGCACAGTGAAACATAGGA		Reverse	GCCGCTGTTGGTGATTTTGT
*TNFRSF1A*	Forward	CTCTCCCCTCCTCTCTGCTT	*mTOR*	Forward	TTATGGGCAGCAACGGACAT
	Reverse	GGGTTGAGACTCGGGCATAG		Reverse	CTTCTCCCTGTAGTCCCGGA
*CASP10*	Forward	CAGGGGCAGGAAGAGAACAG	*JNK1*	Forward	ACATTGAGCAGAGCAGGCAT
	Reverse	ACTAGGAAACGCTGCTCCAC		Reverse	GTCAGGAGCAGCACCATTCT
*CASP8*	Forward	TATCCCGGATGGCTGACT	*BCL2*	Forward	GGAGCGTCAACAGGGAGATG
	Reverse	GACATCGCTCTCAGGCTC		Reverse	GATGCCGGTTCAGGTACTCAG
*SMAD2*	Forward	GGCCTCACGTCATCTACTGCC	*BBC3*	Forward	ATGCCTGCCTCACCTTCATC
	Reverse	GGGTTACGGAA-GCGTGGCAGCAT		Reverse	TCAGCCAAAATCTCCCACCC
*CDK6*	Forward	CGGGATCCACCATGGAGAAGGACGGCCTG	*BAX*	Forward	AGTAACATGGAGCTGCAGAGG
	Reverse	CGGATCCATTGCTCAGGCTGTATTCAGCTCCGA		Reverse	ATGGTTCTGATCAGTTCCGG
*CDK2*	Forward	CTTTGGAGTCCCTGTCCGTA	*BAD*	Forward	AGAGTTTGAGCCGAGTGAGC
	Reverse	CGAAAGATCCGGAAGAGTTG		Reverse	CATCCCTTCGTCGTCCTCC
*TNFRSF10B*	Forward	CGTCCGCATAAATCAGCACG	*β-actin*	Forward	TGTGATGGTGGGAATGGGTCAG
	Reverse	TCTGTCCCCGTTGTTCCATG		Reverse	TTTGATGTCACGCACGATTTCC

## Data Availability

All the data and information related to this study are described elaborately in the paper and Supplementary Materials.

## Supplementary Materials

The following supporting information can be downloaded at https://www.mdpi.com/article/10.3390/molecules31020214/s1, Figure S1: Original Western blot figures of cell cycle related proteins such as (a) β-actin, (b) CDK2, (c) p16, (d) p21, and (e) Cyclin B, corresponding to the data provided in the main text. Figure S2: Original Western blot figures of mitochondria pathway related proteins such as (a) Bax, (b) Bad, (c) Puma, (d) Cyto-c, (e) Casp-3 and (f) Casp-9, corresponding to the data provided in the main text. Figure S3: Original Western blot figures of death receptor related proteins such as (a) Casp-10, and (b) Casp-10, corresponding to the data provided in the main text.

## References

[1] American Cancer Society (2022). Global Cancer Facts & Figures 4th Edition. https://www.cancer.org/research/cancer-facts-statistics/global-cancer-facts-and-figures.html.

[2] Bray F., Laversanne M., Sung H., Ferlay J., Siegel R.L., Soerjomataram I., Jemal A. (2024). Global cancer statistics 2022: GLOBOCAN estimates of incidence and mortality worldwide for 36 cancers in 185 countries. CA A Cancer J. Clin..

[3] Ma Y.-L., Zhang Y.-S., Zhang F., Zhang Y.-Y., Thakur K., Zhang J.-G., Wei Z.-J. (2019). Methyl protodioscin from Polygonatum sibiricum inhibits cervical cancer through cell cycle arrest and apoptosis induction. Food Chem. Toxicol..

[4] Sekar P.K.C., Thomas S.M., Veerabathiran R. (2024). The future of cervical cancer prevention: Advances in research and technology. Explor. Med..

[5] Ahmad B., Khan S., Nabi G., Gamallat Y., Su P., Jamalat Y., Duan P., Yao L. (2019). Natural gypenosides: Targeting cancer through different molecular pathways. Cancer Manag. Res..

[6] Bokelmann J.M. (2022). Gynostemma/Jiaogulan (Gynostemma Pentaphyllum).

[7] Quang H.T., Thi P.T.D., Sang D.N., Tram T.T.N., Huy N.D., Dung T.Q., The Q.T.T. (2022). Effects of Plant Elicitors on Growth and Gypenosides Biosynthesis in Cell Culture of *Giao co lam* (*Gynostemma pentaphyllum*). Molecules.

[8] Wang J., Yang J.-L., Zhou P.-P., Meng X.-H., Shi Y.-P. (2017). Further New Gypenosides from Jiaogulan (*Gynostemma pentaphyllum*). J. Agric. Food Chem..

[9] Wang Z.-X., Yang J.-M., Zhang Z.-B., Zhu X.-T., Xiang P., Sun J., He X.-H. (2023). Chemical constituents and biological activity of *Gynostemma pentaphyllum*: A review. J. South. Agric..

[10] Ji X., Shen Y., Guo X. (2018). Isolation, structures, and bioactivities of the polysaccharides from *Gynostemma pentaphyllum* (Thunb.) Makino: A review. BioMed Res. Int..

[11] Su C., Li N., Ren R., Wang Y., Su X., Lu F., Zong R., Yang L., Ma X. (2021). Progress in the medicinal value, bioactive compounds, and pharmacological activities of *Gynostemma pentaphyllum*. Molecules.

[12] Ahmed A., Saleem M.A., Saeed F., Afzaal M., Imran A., Nadeem M., Ambreen S., Imran M., Hussain M., Al Jbawi E. (2023). *Gynostemma pentaphyllum* an immortal herb with promising therapeutic potential: A comprehensive review on its phytochemistry and pharmacological perspective. Int. J. Food Prop..

[13] Anuradha B., Vaibhav C. *Gynostemma pentaphyllum* Extract Market: Trends & Growth Analysis. Market Research Report, 2024. https://www.wiseguyreports.com/reports/gynostemma-pentaphyllum-extract-market.

[14] Yuge N., Wei Y., Junli L., Wenbing Y., Liangli Y. (2013). Characterization of a Novel Polysaccharide from Tetraploid *Gynostemma pentaphyllum* Makino. J. Agric. Food Chem..

[15] Deng Q., Yang X. (2014). Protective effects of *Gynostemma pentaphyllum* polysaccharides on PC12 cells impaired by MPP(+). Int. J. Biol. Macromol..

[16] Ahn Y., Lee H.S., Lee S.H., Joa K.L., Lim C.Y., Ahn Y.J., Hong K.B. (2023). Effects of gypenoside L-containing *Gynostemma pentaphyllum* extract on fatigue and physical performance: A double-blind, placebo-controlled, randomized trial. Phytother. Res..

[17] Li Y., Ouyang Q., Li X., Alolgal R.N., Fan Y., Sun Y., Ma G. (2023). The role of *Gynostemma pentaphyllum* in regulating hyperlipidemia. Am. J. Chin. Med..

[18] Ren D., Zhao Y., Zheng Q., Alim A., Yang X. (2019). Immunomodulatory effects of an acidic polysaccharide fraction from herbal *Gynostemma pentaphyllum* tea in RAW264.7 cells. Food Funct..

[19] Zhang Z., Zhang W., Ji Y.P., Zhao Y., Wang C.G., Hu J.F. (2010). Gynostemosides A-E, megastigmane glycosides from *Gynostemma pentaphyllum*. Phytochemistry.

[20] Liu H.L., Kao T.H., Chen B.H. (2004). Determination of Carotenoids in the Chinese Medical Herb Jiao-Gu-Lan (*Gynostemma pentaphyllum* MAKINO) by Liquid Chromatography. Chromatographia.

[21] Huang S., Hung C., Wu W., Chen B. (2008). Determination of chlorophylls and their derivatives in *Gynostemma pentaphyllum* Makino by liquid chromatography–mass spectrometry. J. Pharm. Biomed. Anal..

[22] Wang X.W., Zhang H.P., Chen F., Wang X., Wen W.Y. (2009). A new lignan from *Gynostemma pentaphyllum*. Chin. Chem. Lett..

[23] Yang X., Zhao Y., Yang Y., Ruan Y. (2008). Isolation and characterization of immunostimulatory polysaccharide from an herb tea, *Gynostemma pentaphyllum* Makino. J. Agric. Food Chem..

[24] Bai M.-S., Gao J.-M., Fan C., Yang S.-X., Zhang G., Zheng C.-D. (2010). Bioactive dammarane-type triterpenoids derived from the acid hydrolysate of *Gynostemma pentaphyllum* saponins. Food Chem..

[25] Yan H., Wang X., Wang Y., Wang P., Xiao Y. (2014). Antiproliferation and anti-migration induced by gypenosides in human colon cancer SW620 and esophageal cancer Eca-109 cells. Hum. Exp. Toxicol..

[26] Yin Q., Chen H., Ma R.-H., Zhang Y.-Y., Liu M.-M., Thakur K., Zhang J.-G., Wei Z.-J. (2021). Ginsenoside CK induces apoptosis of human cervical cancer HeLa cells by regulating autophagy and endoplasmic reticulum stress. Food Funct..

[27] Tavakoli F., Jahanban-Esfahlan R., Seidi K., Jabbari M., Behzadi R., Pilehvar-Soltanahmadi Y., Zarghami N. (2018). Effects of nano-encapsulated curcumin-chrysin on telomerase, *MMPs* and *TIMPs* gene expression in mouse B16F10 melanoma tumour model. Artif. Cells Nanomed. Biotechnol..

[28] Xing S.F., Liu L.H., Zu M.L., Ding X.F., Cui W.Y., Chang T., Piao X.L. (2018). The inhibitory effect of gypenoside stereoisomers, gypenoside L and gypenoside LI, isolated from *Gynostemma pentaphyllum* on the growth of human lung cancer A549 cells. J. Ethnopharmacol..

[29] Wang T.X., Shi M.M., Jiang J.G. (2018). Bioassay-guided isolation and identification of anticancer and antioxidant compounds from *Gynostemma pentaphyllum* (Thunb.) Makino. RSC Adv..

[30] Hussain S.S., Zhang F., Zhang Y., Thakur K., Naudhani M., Cespedes-Acuña C.L., Wei Z. (2020). Stevenleaf from *Gynostemma Pentaphyllum* inhibits human hepatoma cell (*HepG2*) through cell cycle arrest and apoptotic induction. Food Sci. Hum. Wellness.

[31] Darzynkiewicz Z. (2011). Recent Advances in Cytometry.

[32] Crowley L.C., Marfell B.J., Scott A.P., Waterhouse N.J. (2016). Measuring cell death by propidium iodide uptake and flow cytometry. Cold Spring Harb. Protoc..

[33] Thompson C.B. (1995). Apoptosis in the pathogenesis and treatment of disease. Science.

[34] Newman D.J., Cragg G.M. (2020). Natural products as sources of new drugs over the nearly four decades from 01/1981 to 09/2019. J. Nat. Prod..

[35] Green D.R., Kroemer G. (2004). The pathophysiology of mitochondrial cell death. Science.

[36] Massagué J. (2004). G1 cell-cycle control and cancer. Nature.

[37] Karimian A., Ahmadi Y., Yousefi B. (2016). Multiple functions of p21 in cell cycle, apoptosis and transcriptional regulation after DNA damage. DNA Repair.

[38] Wang J., Liao A.-M., Thakur K., Zhang J.-G., Huang J.-H., Wei Z.-J. (2019). Licochalcone B extracted from *Glycyrrhiza uralensis* fisch induces apoptotic effects in human hepatoma cell *Hepg2*. J. Agric. Food Chem..

[39] Serrano M., Hannon G.J., Beach D. (1993). A new regulatory motif in cell-cycle control causing specific inhibition of cyclin D/*CDK4*. Nature.

[40] Ruas M., Peters G. (1998). The p16INK4a/*CDKN2A* tumor suppressor and its relatives. Biochim. Biophys. Acta-Rev. Cancer.

[41] Fan J.P., Kim H.S., Han G.D. (2009). Induction of apoptosis by l-carnitine through regulation of two main pathways in Hepa1c1c 7 cells. Amino Acids.

[42] McCoy M.K., Tansey M.G. (2008). TNF signaling inhibition in the CNS: Implications for normal brain function and neurodegenerative disease. J. Neuroinflamm..

[43] Siddiqui W.A., Ahad A., Ahsan H. (2015). The mystery of BCL2 family: Bcl-2 proteins and apoptosis: An update. Arch. Toxicol..

[44] Pan Z., Zhang X., Yu P., Chen X., Lu P., Li M., Liu X., Li Z., Wei F., Wang K. (2019). Cinobufagin induces cell cycle arrest at the G2/M phase and promotes apoptosis in malignant melanoma cells. Front. Oncol..

[45] Sun Y.-S., Thakur K., Hu F., Zhang J.-G., Wei Z.-J. (2020). Icariside II inhibits tumorigenesis via inhibiting *AKT*/Cyclin E/*CDK 2* pathway and activating mitochondria-dependent pathway. Pharmacol. Res..

[46] Wang J., Zhang Y.-S., Thakur K., Hussain S.S., Zhang J.-G., Xiao G.-R., Wei Z.-J. (2018). Licochalcone A from licorice root, an inhibitor of human hepatoma cell growth via induction of cell apoptosis and cell cycle arrest. Food Chem. Toxicol..

