# Supplementary material for: From Functional Food to Therapeutic Prospect: Mechanistic Study of Gypenoside XVII in HeLa Cells
Source: Molecules. 2026 Jan 8;31(2):214. doi: 10.3390/molecules31020214 (PMC12843943; doi:10.3390/molecules31020214)
Supplement: Supplementary file 1 [file molecules-31-00214-s001.zip › molecules-4059439-SI.pdf]

Raw Data Associated with Uncropped Western Blots

The supporting information contains the raw data of all uncropped western blot images related to cell cycle related proteins, mitochondria pathway related proteins, and death receptor pathway related proteins associated with the manuscript "From Functional Food to Therapeutic Prospect: Mechanistic Study of Gypenoside XVII in HeLa Cells."

Cell Cycle Related Proteins

β-actin used as a control. And the mentioned band (1, 2, 3, 4) described treated Hela cell with different concentration of gypenoside XVII, such as 1. Untreated Hela cells, 2. Treated Hela cells with 90 μM gypenoside XVII, 3. Treated Hela cells with 130 μM gypenoside XVII, and 4. Treated Hela cells with 150 μM gypenoside XVII.

Figure. S1 (a) β-actin

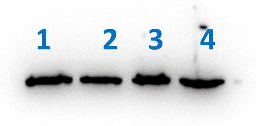

Figure. S1 (b) CDK2

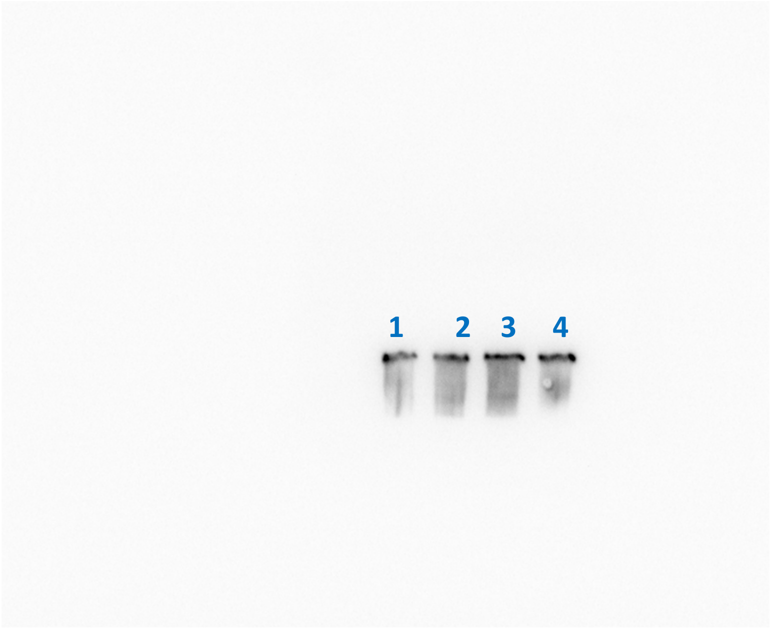

Figure. S1 (c) p16

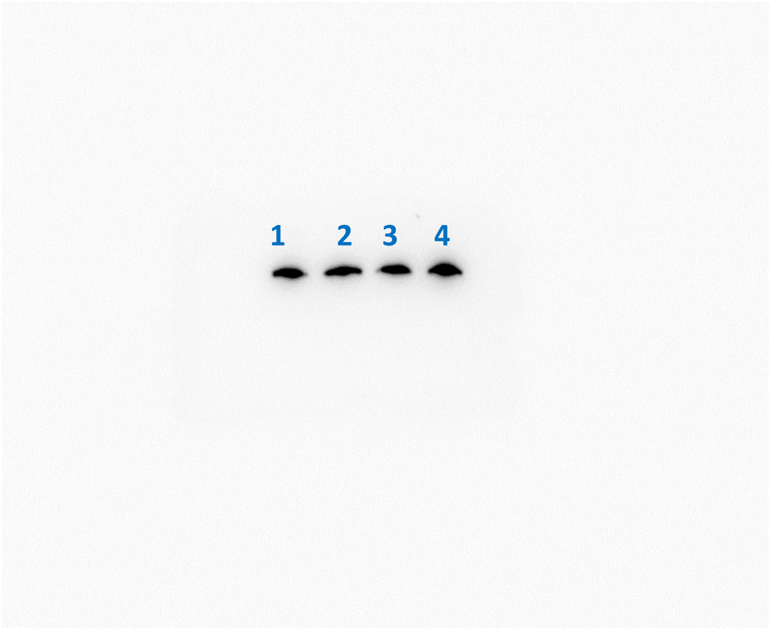

Figure. S1 (d) p21

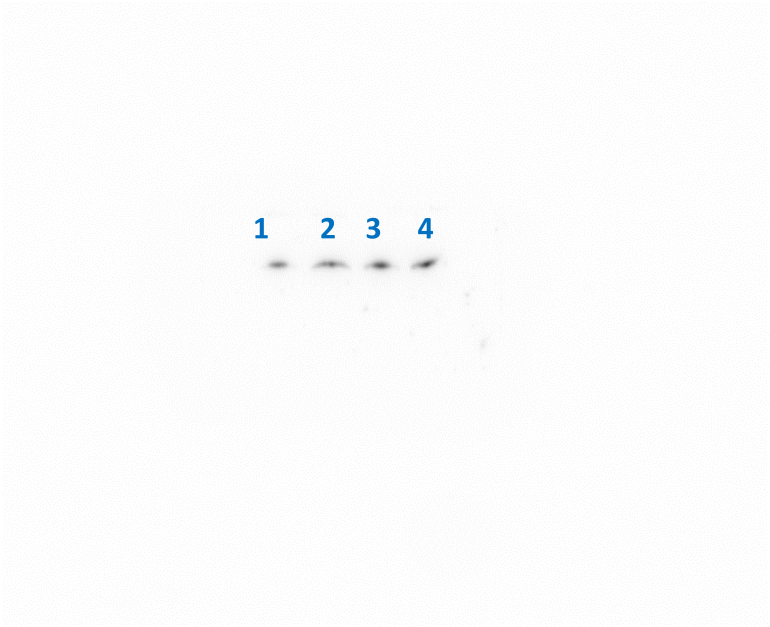

Figure. S1 (e) Cyclin B

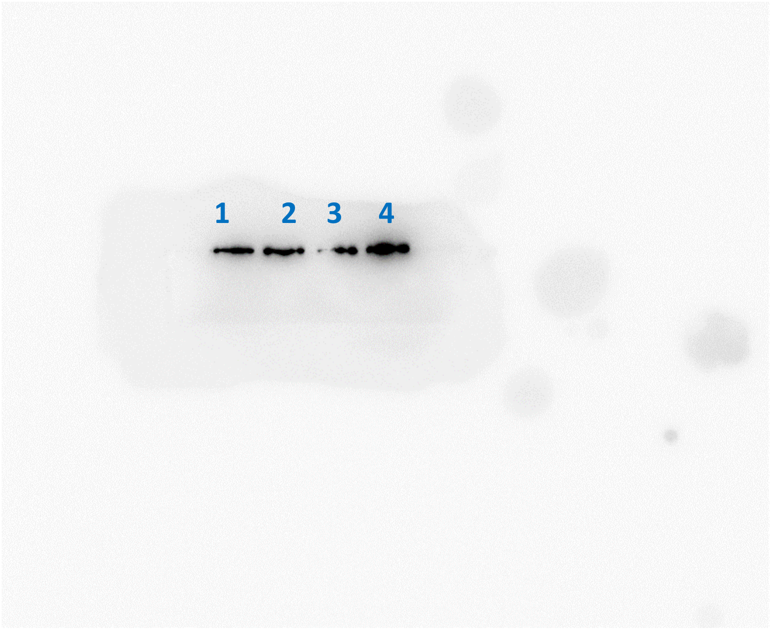

Figure S1: Original Western blot figures of cell cycle related proteins such as (a) β-actin, (b) CDK2, (c) p16, (d) p21, and (e) Cyclin B, corresponding to the data provided in the main text.

Mitochondria Pathway Related Proteins

Figure. S2 (a) Bax

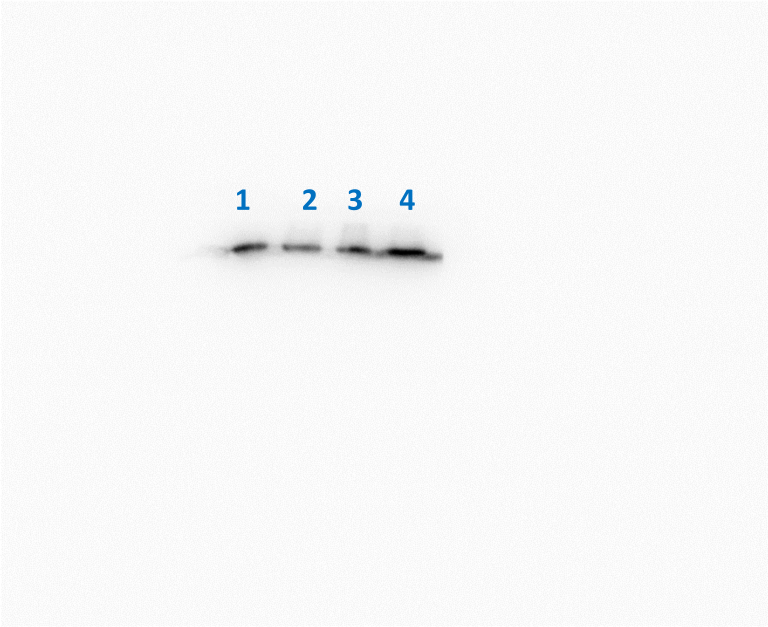

Figure. S2 (b) Bad

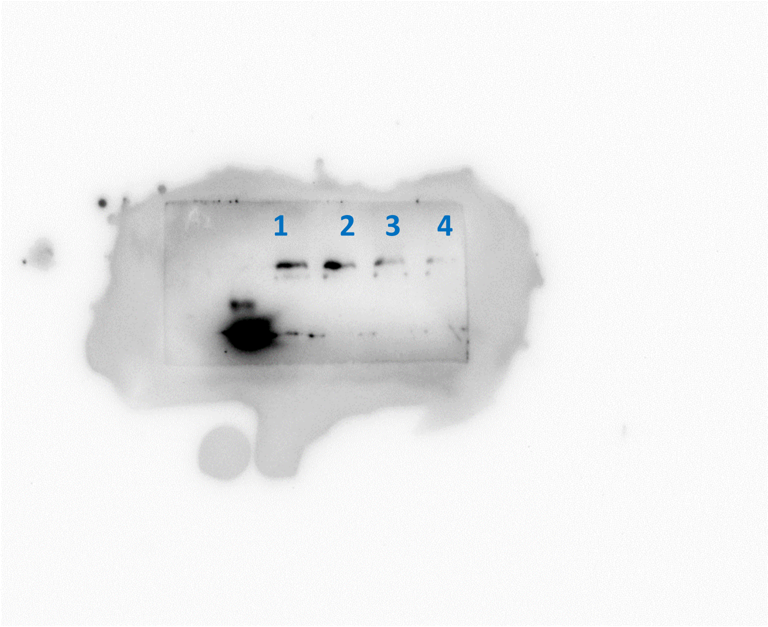

Figure. S2 (c) PUMA

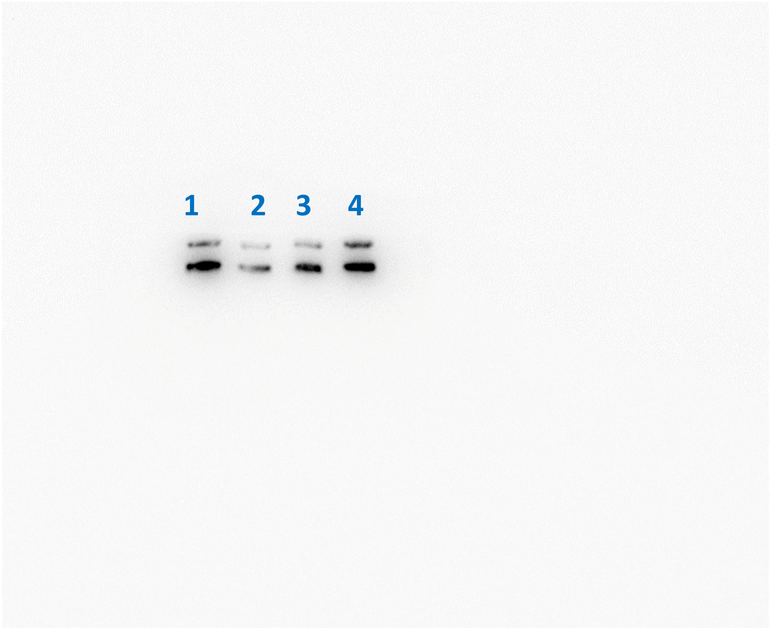

Figure. S2 (d) Cyto-c

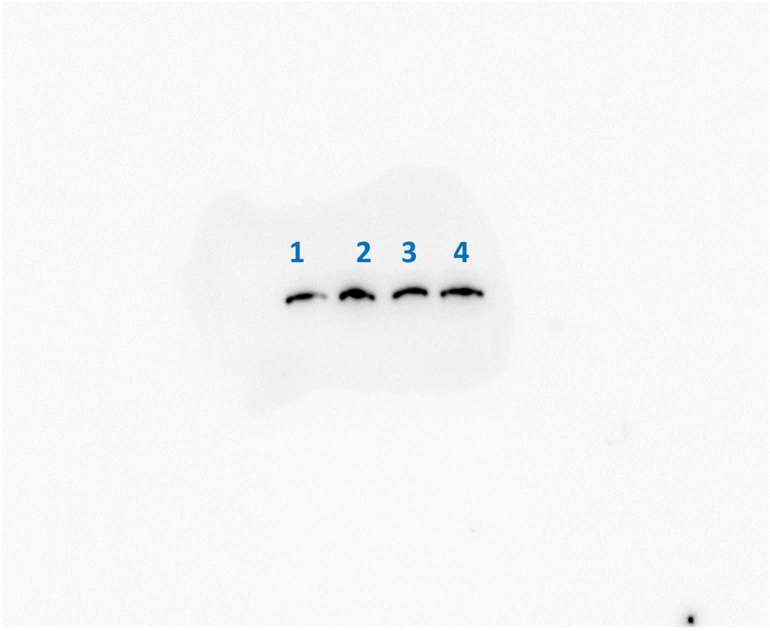

Figure. S2 (e) Caspse-3

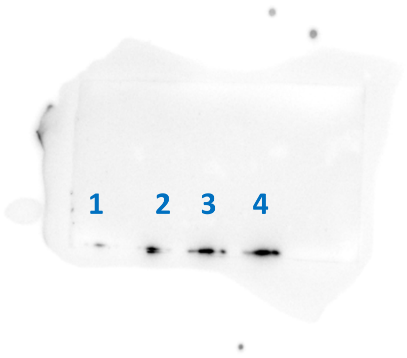

Figure. S2 (f) Caspase-9

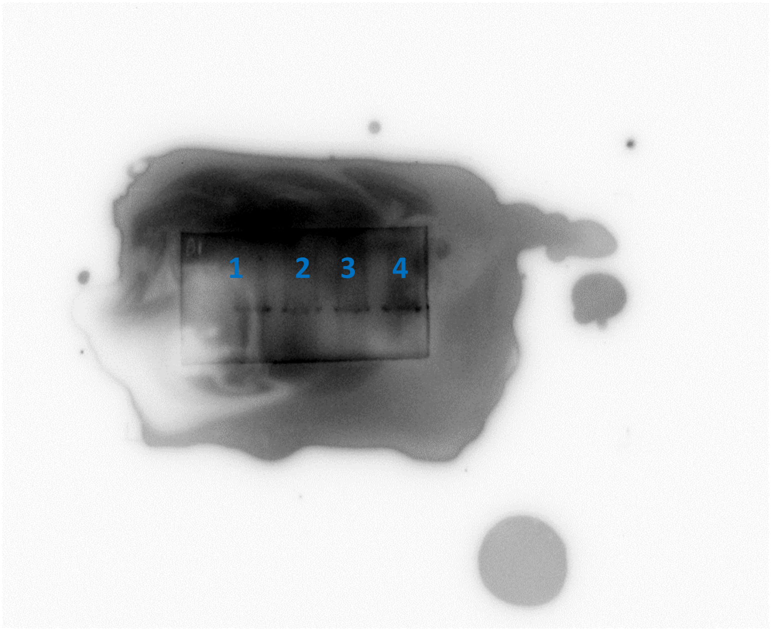

Figure S2 : Original Western blot figures of mitochondria pathway related proteins such as (a) Bax, (b) Bad, (c) Puma, (d) Cyto-c, (e) Casp-3 and (f) Casp-9, corresponding to the data provided in the main text.

Death Receptor Pathway Related Proteins

Figure. S3 (a) Caspase-10

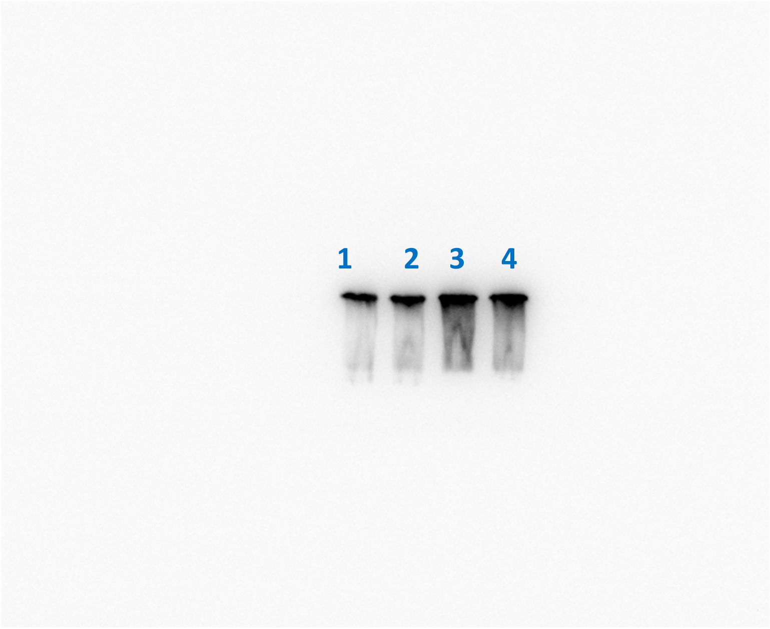

Figure. S3 (b) Caspase-8

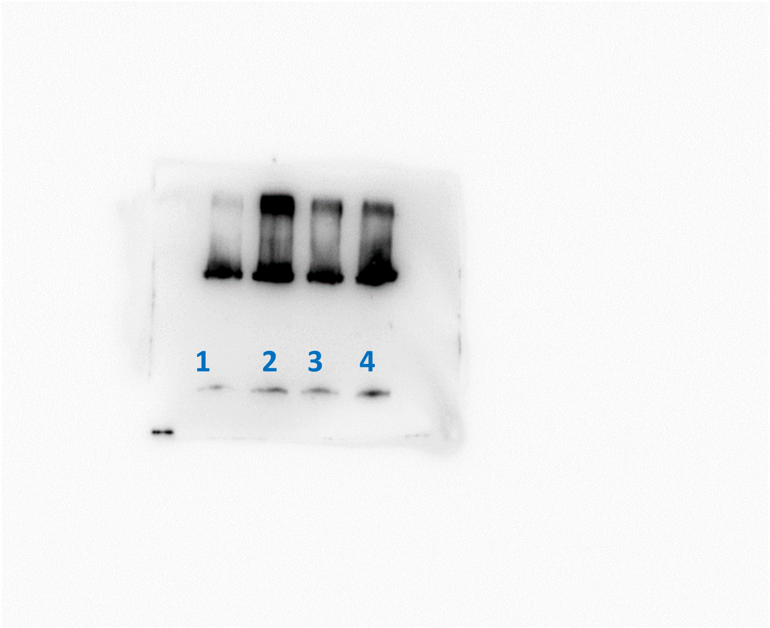

Figure S3: Original Western blot figures of death receptor related proteins such as (a) Casp-10, and (b) Casp-10, corresponding to the data provided in the main text.
